# Cystic Echinococcosis: Knowledge, Attitude, and Practices (KAP) among Surgically Operated Cases in Fars Province, Southern Iran

**DOI:** 10.1155/2021/9976548

**Published:** 2021-04-08

**Authors:** Zahra Hosseini, Reza Shahriarirad, Bahador Sarkari

**Affiliations:** ^1^Department of Parasitology and Mycology, School of Medicine, Shiraz University of Medical Sciences, Shiraz, Iran; ^2^Student Research Committee, Shiraz University of Medical Sciences, Shiraz, Iran; ^3^Basic Sciences in Infectious Diseases Research Center, Shiraz University of Medical Sciences, Shiraz, Iran

## Abstract

**Introduction:**

Cystic echinococcosis (CE) is a neglected zoonotic disease caused by *Echinococcus granulosus* with major health and economic burden. The information on how the community members perceive the disease is crucial in order to recommend an effective preventive and control plan. The current study is aimed at finding out knowledge, attitude, and practices (KAP) of surgically operated cases of hydatid cyst in educational hospitals of Shiraz in Fars Province, southern Iran, toward the CE.

**Methods:**

A cross-sectional survey was conducted among 180 CE patients who underwent surgery due to CE. Using a well-designed questionnaire, a telephone-based survey was carried out to collect the data. The contents of the questionnaire included basic personal information and questions related to the participants' knowledge, attitude, and behavioral patterns toward CE. Univariate and then multivariate linear regression analyses were used to identify factors associated with the KAP. Unstandardized regression coefficients (*β*) and odds ratios (ORs) and their 95% confidence intervals (CIs) were used to quantify the associations between variables and KAP.

**Results:**

A total of 180 CE patients with a mean age of 35.64 (±17.59) years were recruited. The mean score of participant's knowledge was 8.7 (SD = 2.8, range: 0-17), whereas these scores were 1.3 (SD = 0.7, range 0-2) for attitude and 1.2 (SD = 1.0, range 0-4) for practice. Findings of the study demonstrated that 20 of the participants (11.1%) had good knowledge towards CE, 82 (45.6%) demonstrated a positive attitude, and 57 (47.5%) without having dogs demonstrated a good practice towards CE (score = 2/2), while from 60 dog owners, only 7 (11.6%) participants demonstrated good practice (score 3 and 4/4). Factors that were associated with knowledge were age (OR = −0.49, *P* value = 0.001) and educational level (OR = 0.668, *P* value = 0.001), where higher age was associated with lower knowledge and also higher educational levels were associated with higher knowledge regarding hydatid cyst. Regarding attitude, only living location had a significant association with participants' attitude where those who were living in urban areas demonstrating a more positive attitude towards CE (OR = 0.261, *P* value = 0.022). The practice of the participants was grouped into dog owners and participants with no dogs, in which among participants who did not own a dog, those living in urban areas demonstrating weaker practice towards CE (OR = −0.491, *P* value>0.001). Moreover, a lack of counseling of patients after the surgery on how to prevent reinfection was noticed.

**Conclusion:**

Findings of the study revealed that the CE patients in southern Iran had poor knowledge and attitude toward the disease, and their practice may help in maintaining the disease in the community. Health education is highly needed to increase community awareness and to prevent and control this neglected parasitic infection in the area.

## 1. Introduction

Cystic echinococcosis (CE) or hydatid cyst is an infectious disease that affects both humans and animals. Livestock (especially sheep) serves as intermediate hosts for the transmission of CE, while canids, mainly dog, act as the definitive hosts. Human acquires the disease by accidental ingestion of eggs existing in dog feces [1, 2].

CE is a major health and economic challenge in the Middle East countries, including Iran where about 1% of all hospital surgeries are accounted for this disease [3–5]. The overall seroprevalence of CE in Iran is reported to be 4.2% with the highest prevalence in the South area with 5.8% and the least in the central area with 2.2% prevalence rate [6]. CE is one of the most important parasitic diseases in Fars Province in the south of Iran [5, 7, 8]. Fars Province is the center of agriculture and animal husbandry in Iran, and one of the most important and populated tribal nomads (Qashqai) resides in this area. The province is endemic focus for several parasitic diseases including visceral and cutaneous leishmaniasis [9, 10]. In a study by Shahriarirad et al. in Fars Province, a total of 501 CE surgical cases were recorded during 15 years, corresponding to an average annual incidence of 33.4 and a surgical incidence rate of 0.74/100,000 population [5].

To introduce and implement effective control measures, knowing the disease mode of transmission and its characteristics is crucial. According to the KAP theory, the controlling of the disease in the community is generally affected by people's knowledge, attitude, and practices (KAP) concerning the disease [11, 12]. Public education is considered as one of the most important measures that can help control the CE, as has been the case in other diseases [13]. Hence, with CE prevention and control programs being a necessity, a well-enough knowledge of the disease accompanied by community cooperation can play a significant role in the success of these programs. The current study is aimed at determining the level of knowledge, attitude, and practices of patients who underwent surgery for hydatid cyst in Fars Province, southern Iran, about the risk factors for hydatid cyst.

## 2. Materials and Methods

### 2.1. Study Design and Participants

In this cross-sectional study, a list of hospitalized CE patients during a period of 6 years (2014–2020) was obtained at the main university-affiliated and referral hospitals in Shiraz, capital of Fars Province. Sample size estimation was done by the Med-Calc statistical software with an error of 5% and a power of 90%. The recruitment method was voluntary participation, and individuals unwilling to participate were excluded from the study. Also, subjects were assured that the results would be reviewed as a group and that the confidentiality of their responses would be preserved.

Only cases with a final diagnosis of any type of CE at hospital discharge which were recorded with a unique disease code (based on the ICD-9 and ICD-10: International Classification of Diseases; 122.9 and 122.8 for ICD9; B67.8, K77.0, B67.9, and J99.8 for ICD10) were included in the data analysis. The data collection tool in this study consisted of a standard and research designed questionnaire, in which each patient was contacted by telephone call and was asked a series of questions by the interviewer.

### 2.2. Ethical Consideration

The study was approved by the Ethics Committee of Shiraz University of Medical Sciences (Ref. No. IR.sums.med.rec.1399.419). Participants who verbally consented to be included in our study were enrolled.

### 2.3. Data Collection

Data collection was done through a researcher-developed questionnaire in which consisted of the following sections:

1. Demographic variables including gender, age, province of residence, educational level, and occupation along with other factors regarding the patient's history of CE
2. Knowledge, attitude, and practice questions, which consisted of 18 questions assessing the participants' KAP towards CE
3. Seven additional questions including “Do you keep dogs in your area?” “Do you own livestock? (cattle, cow, sheep)” “Have you ever seen a hydatid cyst in an animal's liver, lungs, or abdomen?” “Did your doctor or nurse give you information about hydatid cysts?” “Has anyone in your family been infected with hydatid cyst” “How severe or fatal do you think this disease is?” “What is your source of information about hydatid cysts?”

A correct answer was assigned as one point while an incorrect answer received no points.

### 2.4. Data Analysis

All the statistical analyses were performed, using Microsoft Excel 2007 (Microsoft Corp., Redmond, USA) and statistical package for social sciences (SPSS Inc., Chicago, Illinois, USA, version 26.0). Univariate and then multivariate linear regression analyses were used to identify factors associated with participants' KAP. Unstandardized regression coefficients (*β*) and odds ratios (ORs) and their 95% confidence intervals (CIs) were used to quantify the associations between variables and KAP. The statistical significance level was set at *P* < 0.05.

## 3. Results

A total of 180 CE patients were recruited in this study and contacted regarding their KAP towards CE. The mean age of the participants was 35.64 (±17.59).  Table 1 demonstrates the demographic features of the participants in our study.

The mean score of the participants' knowledge toward CE was 8.7 (± 2.8, range: 0-17), the mean score of attitude was 1.3 (± 0.7, range 0-2), and the mean score of practice was 1.2 (±1.0, range 0 - 4) for dog owners and 1.4 (±0.7, range 0-2) for participants who did not own dogs.  Table 2 shows the details of participants' KAP toward CE, based on the questions asked.

To further review and analyze the findings, the KAP of the participants were categorized into good, moderate, and weak. The knowledge score (ranging from 0-17) was grouped as score range of 0-5 as weak/low knowledge, 6–11 as moderate knowledge, and 12-17 as good knowledge towards CE. The findings demonstrated that 23 (12.8%) of the participants had low knowledge, while 137 (76.1%) had moderate knowledge, and 20 (11.1%) had good knowledge of CE.

Attitude score ranged from 0 to 2. Based on the results of our study, 82 (45.6%) of the participants demonstrated a positive attitude towards CE, while 72 (40%) demonstrated moderate, and 26 (14.4%) demonstrated a negative attitude towards CE. Based on the participants' practice score, 57 (47.5%) of the participants without dogs demonstrated a good practice towards CE (score = 2/2), while 50 (41.7%) of them demonstrated moderate (score = 1/2) and 13 (10.8%) demonstrated a weak practice towards CE (score = 0/2). Regarding the 60 dog owners, only 7 (11.6%) demonstrated good practice (score 3 and 4/4), while 12 (20%) demonstrated moderate practice (score = 2/4), and 41 (68.3%) demonstrated weak practice (score = 0 and 1/4) towards CE.

To evaluate the effect of the variables of our study in the participants' KAP, multiple linear regression analysis was used, and the results are given in Table 3.

As demonstrated in Table 3, factors that were associated with knowledge were age (OR = −0.49, *P* value = 0.001) and educational level (OR = 0.668, *P* value = 0.001), where higher age was associated with lower knowledge and also higher educational levels were associated with higher knowledge regarding hydatid cyst. Regarding attitude, only living location had a significant association, with participants living in urban areas demonstrating a more positive attitude towards CE (OR = 0.261, *P* value = 0.022).

The participants were also asked a series of additional questions, which are reported in Table 4. The sources of information for most of the participants have been social media and the internet. Moreover, a lack of counseling of patients after the surgery on how to prevent CE reinfection was noticed.

## 4. Discussion

In the current study, the KAP of CE patients towards the disease was assessed. As our results show, knowledge, attitude, and practice of the CE patients were relatively low with only 11.1% of the participants demonstrating good knowledge, 45.6% demonstrating a positive attitude, and 11.6% of dog owners and 47.5% of participants with no dog demonstrating suitable practice towards CE.

Our results are similar to the findings of Abdulhameed et al., a study on surgical cases of CE in Iraq, which reported that 72% of the participants had not heard of CE and 57% did not know the mode of transmission of hydatid cysts, even after surgery for the disease [14].

In a community based cross-sectional study by Dan Li et al. [1] in Xiahe County, in China, 65.9% of the participants have heard the name of CE. A study in Pakistan by Khan et al. [15], regarding the knowledge, attitudes, and practices on the occurrence of CE in butchers and dog owners in both urban and rural areas of Rawalpindi/Islamabad regions, showed that only 4.1% of people have heard about the disease. In a study regarding KAP towards CE in China by Yin et al. [16], the findings indicated that the KAP rate for all participants was 72.6%, 6.4%, 95.0%, and 75.8% for KAP, *K*, *A*, and *P*, respectively. The KAP rates were significantly different among different age and gender groups.

In a study in Ethiopia [17] on risk factors on public awareness, attitude, and practices of CE, only 4.29% of pastoralists responded that they were aware of the occurrence of the disease in human, but none of them were knowledgeable on its sources and transmission.

A cross-sectional survey by Ahmed et al. [18], in three villages around the city of Tambool in Central Sudan, showed that 68.7% of the participants had never heard of the disease.

A study in Turkey [19] on 151 farmers regarding KAP towards CE showed that the knowledge level of livestock farmers is very low (21.9%). A study by Ozlu et al. [20] on 1,045 cattle farmers in Erzurum in Turkey showed that the increase in education status, size of the enterprise, and monthly income of cattle farmers were related to an increase in knowledge, attitude, and practices regarding zoonotic diseases. However, it was found that the positive knowledge and attitudes of the cattle farmers could not be transformed into positive practices evenly.

Singh et al. [21] conducted a study to find out the knowledge, attitude, and practices of livestock farmers regarding zoonoses in India where the low level of education and being a cattle farmer were negatively associated with the farmer's knowledge on zoonotic diseases.

Our results are also in line with the findings of other questionnaire surveys conducted in Libya and Morocco [22, 23], which found that the majority of respondents had limited to no information about the transmission of CE. Increasing knowledge and information regarding diseases is an essential step towards the management and prevention of the disease.

Among the noteworthy results of our study is that only 11.6% of dog owners demonstrated suitable practice towards CE. Studies from southern Iran regarding free-roaming and stray dogs reported a prevalence of 10.7% to 36.19% for *E. granulosus* infection in dogs [24, 25]. The close contact of people with dogs, combined with feeding offal, along with environmental contamination enhances the likelihood of transmission of this zoonotic tapeworm to humans, especially the children [7, 26, 27].

In our study, 62.5% of the participants reported slaughtering and butchering at home. Abdulhameed et al. reported that around 50% of surgical cases of CE in Iraq performed home slaughtering and butchering [14]. Many studies have highlighted the effect of the common practice of slaughtering animals by householders in or near their homes on CE infection [28, 29]. It is obvious that under the supervision of a veterinarian, the slaughter of animals in a slaughterhouse reduces the ability to complete the life cycle of *Echinococcus* by ensuring proper disposal of infected offal [30].

In partnership with the veterinary authorities, the health authorities in Iran and also in any CE-endemic areas need to establish and introduce educational programs on echinococcosis for farmers, pet owners, and the general population. These initiatives should include information on the need for deworming of dogs; better hygiene for food preparation; animal slaughter at home, including strict instructions on how to extract infected offal; how to disrupt the life cycle of *Echinococcus* and how to avoid its development; and practices to reduce the dog infection.

## 5. Conclusion

Findings of the current study revealed that the CE patients in southern Iran had poor knowledge and attitude toward the disease, and their improper practice may help in maintaining the disease in the community. Successful education and awareness programs in the community for the prevention and control of CE can be of great benefit. Improving the degree of awareness of the community about CE is the groundwork for the prevention and management of the diseases which in turn improves the attitude of people in the community.

## Figures and Tables

**Table 1:** Demographic features of participants in the study.

Variable	Frequency	
	Number	Percentage
Age group		
10-20	42	23.3
21–30	39	21.7
31–40	28	15.6
41–50	27	15
51-60	28	15.6
> 60	16	8.9
Gender		
Male	91	50.6
Female	89	49.4
Occupation		
Unemployed	102	56.7
Self-employed	43	23.9
Freelance	14	7.8
Retired	8	4.4
Nongovernmental employee	7	3.9
Student	4	2.2
Government employee	2	1.1
Educational level		
Illiterate	17	9.4
Reading and writing	25	13.9
Under high school diploma	78	43.3
Diploma	42	23.3
University degree	18	10
Residence		
Urban	88	48.9
Rural	92	48.9
Marital status		
Single	72	40
Married	108	60
Number of individuals in the household		
<5	118	65.6
≥ 5	62	34.4
Living location		
Apartment	21	11.7
House/villa	159	88.3
Year of operation for CE		
2020	51	28.3
2019	58	32.2
2018	35	19.4
2017	30	16.7
2016	5	2.8
2014	1	0.6
Location of CE		
Lung	118	65.6
Liver	59	32.8
Other locations	3	1.7
Number of CE		
One	68	41
Two	58	34.9
More than 2	40	24.1

**Table 2:** Questionnaire items and responses regarding KAP among participants with a history of hydatid cyst surgery.

Question	Answers	Frequency	
		Number	Percentage
Knowledge			
(1) Have you had heard about hydatid cyst before you were infected with it?			
	Yes^∗^	8	4.4
	No	172	95.6
(2) What are the symptoms of a hydatid cyst?			
	Gastrointestinal symptoms^∗^	73	40.6
	Liver symptoms^∗^	41	22.8
	Pulmonary symptoms^∗^	104	57.8
	Cerebral symptoms^∗^	9	5
	Do not know	43	23.9
(3) Do you know which animal is the source and reservoir of hydatid cyst?			
	Dog^∗^	131	72.8
	Cat	15	8.3
	Sheep	9	5
	Do not know	25	13.9
(4) Do you know how a person can get infected with hydatid cyst?			
	Yes^∗^	122	67.8
	No	58	32.2
(5) Do you know what causes hydatid cyst disease?			
	Parasite^∗^	127	70.6
	Microbe	16	8.9
	Virus	1	0.6
	Do not know	36	20
(6) Do you know the route of transmission of hydatid cyst?			
	Yes^∗^	131	72.8
	No	49	27.2
(7) Do you know how to prevent hydatid cysts?			
	Yes^∗^	125	69.4
	No	55	30.6
(8) Do you think this disease is heritable?			
	Yes	18	10
	No^∗^	162	90
(9) To what extent do you think measures can be taken to prevent the disease?^∗^			
	Very high^∗^	93	51.7
	High^∗^	63	35
	Low	21	11.7
	Very low	3	1.7
(10) Did you know that playing with dogs can put you at risk for hydatid cyst disease?			
	Yes^∗^	142	78.9
	No	5	2.8
	Do not know	33	18.3
(11) Is hydatid cyst transmitted to humans through eating the meat or liver of animals (sheep, cattle, goats)?^∗^			
	Yes	131	72.8
	No^∗^	12	6.7
	Do not know	37	20.6
(12) Is hydatid cyst transmitted through vegetables that are not well washed?			
	Yes^∗^	162	90
	No	2	1.1
	Do not know	16	8.9
(13) Can a person who gets hydatid cyst get the disease again?			
	Yes^∗^	74	41.1
	No	31	17.2
	Do not know	75	41.7
(14) Is hydatid cyst transmitted in children through play with soil?			
	Yes^∗^	129	71.7
	No	13	7.2
	Do not know	38	21.1
Attitude			
(15) In your opinion, how much luck is involved in the development of hydatid cysts?			
	A lot	63	35
	Not much^∗^	117	65
(16) In case of infection, how much do you think traditional healers and local remedies can help treat you?			
	Very high	9	5
	High	52	28.9
	Low^∗^	68	37.8
	Very low^∗^	51	28.3
Practice			
(17) What is your source of drinking water?			
	Tap water^∗^	125	69.4
	Well	41	22.8
	Fountain	14	7.8
(18) Do you treat your dog for parasites?			
	Yes^∗^	22	12.2
	No	38	21.1
	Do not own a dog	120	66.7
(19) If you keep dogs at home, do you use the liver, lungs, or other parts of sheep and goats to feed them?			
	Yes	43	23.9
	No^∗^	17	9.4
	Do not own	120	66.7
(20) Do you sometimes butcher at home?			
	Yes	112	62.2
	No^∗^	68	37.8

^∗^ indicates the desired answer.

**Table 3:** Linear regression analysis of factors associated with KAP among participants with a history of hydatid cyst surgery.

Model	Beta	*T*	*P* value	*R*	*R* ^2^
Knowledge				0.433	0.188
Age	-0.49	-3.240	0.001		
Education (illiterate)	0.668	3.309	0.001		
Residence (urban)	-0.378	-0.940	0.349		
Marital status (single)	0.686	1.321	0.188		
Attitude				0.175	0.031
Age	0.001	0.339	0.735		
Education (illiterate)	0.035	0.627	0.531		
Residence (urban)	0.261	2.318	0.022		
Marital status (single)	-0.081	-0.559	0.577		
Practice (no dog)				0.440	0.194
Age	-0.004	-1.030	0.305		
Education (illiterate)	0.032	0.524	0.601		
Residence (urban)	-0.491	-4.174	>0.001		
Marital status (single)	-0.180	-1.215	0.227		
Practice (dog owner)				0.342	0.117
Age	-0.016	-1.306	0.197		
Education (illiterate)	0.070	0.459	0.648		
Residence (urban)	-0.303	-0.971	0.336		
Marital status (single)	0.157	0.392	0.696		

**Table 4:** Additional questions regarding participants' perspective towards CE.

Questions	Frequency	
	Number	Percentage
(1) Do you keep dogs in your area?		
Yes	60	33.3
No	120	66.7
(2) Do you own livestock? (cattle, cow, sheep)		
Yes	66	36.7
No	114	63.3
(3) Have you ever seen a hydatid cyst in an animal's liver, lungs, or abdomen?		
Yes	68	37.8
No	112	62.2
(4) Did your doctor or nurse give you information about hydatid cysts?		
Yes	166	92.2
No	14	7.8
(5) Has anyone in your family been infected with hydatid cyst?		
Yes	5	2.8
No	175	97.2
(6) How severe or fatal do you think this disease is?		
Very high	93	51.7
High	63	35
Low∗	21	11.7
Very low∗	3	1.7
(7) What is your source of information about hydatid cysts?		
Internet and social media	71	39.4
Scientific articles and books	44	24.4
Healthcare professionals and doctors	165	91.7
None	12	6.7

## Data Availability

The nominal and ordinal data used to support the findings of this study are available from the corresponding author upon request.
